# Potential of solar-induced chlorophyll fluorescence for monitoring long-term dynamics of soil salinity in Central Asia the Xinjiang Region China

**DOI:** 10.3389/fpls.2025.1603159

**Published:** 2025-07-09

**Authors:** Kuangda Cui, Jianli Ding, Jinjie Wang, Jiao Tan, Lijing Han, Jiangtao Li

**Affiliations:** ^1^ College of Geography and Remote Sensing Sciences, Xinjiang University, Urumqi, China; ^2^ Xinjiang Key Laboratory of Oasis Ecology, Xinjiang University, Urumqi, China; ^3^ Xinjiang Institute of Technology, Aksu, China; ^4^ College of Geodesy and Geomatics, Shandong University of Science and Technology, Qingdao, China

**Keywords:** soil salt, solar-induced fluorescence, coarse-resolution solar-induced fluorescence, world soil information service, remoting sensing

## Abstract

**Introduction:**

Soil salinization in Central Asia and Xinjiang, China, poses serious threats to agriculture and ecosystems. Solar-induced chlorophyll fluorescence (SIF), which reflects plant photosynthetic status and stress, shows promise for monitoring salinity but remains underutilized in this region.

**Methods:**

This study integrated SIF-derived indices (SIFI) with soil salinity data to build a region-specific prediction model. Using a random forest algorithm, soil salinity was classified into five levels based on satellite data and ground references from 2000–2020. Model performance, seasonal sensitivity, and spatial variation were analyzed across Central Asian countries and Xinjiang.

**Results:**

SIF effectively detected salinization dynamics, with highest sensitivity in Kazakhstan and Xinjiang. April was identified as the most responsive month, with SIFI1 being the key indicator. The model achieved over 80% accuracy in typical regions and around 70% in atypical regions. Kazakhstan had the largest salt-affected area, followed by Turkmenistan and Xinjiang. Tajikistan showed high variability, while Xinjiang remained relatively stable. Most areas exhibited increasing salinity and expansion of saline lands.

**Discussion:**

These findings demonstrate the potential of SIF-based monitoring for large-scale salinity assessment. The integration of plant physiological signals with machine learning provides a valuable tool for early warning and sustainable land management in arid regions.

## 1 Introduction

The issue of soil salinization in Centra (Singh, 2022)l Asia is becoming increasingly severe, posing a significant challenge to sustainable agricultural development and ecological environmental protection in the region. According to a 2015 report by the Food and Agriculture Organization of the United Nations, the area of saline-alkali land in Central Asia has reached 91.5 million hectares, accounting for 20% of the global total, primarily concentrated in Kazakhstan, Uzbekistan, southern Turkmenistan, and the Xinjiang region of China (Khasanov et al., 2023). This phenomenon not only significantly reduces the agricultural productivity of the land but also leads to soil degradation, disrupts the balance of ecosystems, and consequently affects the livelihoods of residents and regional ecological security (Singh, 2022).

The unique climatic conditions of Central Asia and Xinjiang, such as high evaporation rates, limited precipitation, and improper irrigation management, further exacerbate the problem of soil salinization. In Central Asia, salinization is mainly caused by secondary soil salinization and poor water resource management (Arshad et al., 2025). Drought conditions, inadequate irrigation systems, and frequent over-irrigation leading to rising water tables, result in salt accumulation, particularly prominent in the Amu Darya, Syr Darya river basins, and the Aral Sea area (Dong et al., 2024). Salinization reduces the soil fertility in these areas and causes widespread land desertification, severely threatening local food security and economic development (Galvani, 2007). The Xinjiang region, China, also faces severe salinization issues, particularly in the Tarim Basin and Turpan Basin (Zhang et al., 2024a). This phenomenon is linked to Xinjiang’s unique geographical and climatic conditions, such as intense evaporation and arid environments, which further exacerbate the problem (Turdaliev et al., 2023). Furthermore, the salinization in Xinjiang is influenced by changes in land use and ecosystem vulnerability, leading to a decline in agricultural productivity and increasingly severe water scarcity issues (Wang et al., 2022). The negative impacts of soil salinization on local ecosystems, such as vegetation degradation and the reduction of biodiversity, have also attracted widespread attention (Ding et al., 2020). Currently, the management of salinization in Xinjiang and Central Asia faces significant challenges, requiring a comprehensive approach that includes water resource management (Shen and Lein, 2005), agricultural technology improvements, and ecological protection measures. The negative impacts on local ecosystems, such as vegetation degradation and biodiversity reduction, have also attracted widespread attention (Zhuang et al., 2021).

Traditional soil salinization monitoring techniques encompass a range of advanced tools, including Sentinel series satellites, Landsat series satellites, Planet Scope satellites (Tan et al., 2023), and uncrewed aerial vehicles (Tan et al., 2024), among other remote sensing devices. These devices offer observational capabilities at various scales, from global to local. Visible light remote sensing (vis) primarily utilizes the reflective properties of soil or vegetation within the visible light spectrum (400–700 nm) to analyze the extent of soil salinization (Wang et al., 2023). Soil salinization alters the spectral reflectance characteristics of vegetation, particularly in the red and near-infrared bands, where soils with higher levels of salinization typically exhibit characteristic spectral reflectance changes (Adeeb and Al-Timimi, 2021). However, VIS is easily influenced by factors such as cloud cover, vegetation coverage, and surface disturbances, leading to unstable data. Moreover, VIS technology primarily reflects surface soil information, making it difficult to directly obtain data on deeper salinization, which limits its application in salinization detection (Adeeb and Al-Timimi, 2021). The NDVI vegetation index, calculated based on reflectance in visible and near-infrared bands, is susceptible to seasonal variations, particularly during crop fallow periods or natural vegetation growth cycles. Variations in NDVI at different growth stages increase the uncertainty in soil salinization monitoring (Montandon and Small, 2008). Additionally, NDVI is influenced by atmospheric conditions, the spectral properties of soil and vegetation, and sensor characteristics, which increase data uncertainty (Khalesi et al., 2024). These uncertainties limit the application of NDVI in soil salinization monitoring, particularly in areas with bare soil or sparse vegetation (Van Leeuwen et al., 2006).

Solar-induced chlorophyll fluorescence (SIF) is widely used to assess the efficiency of vegetation photosynthesis. This method is based on plants absorbing light energy during photosynthesis and re-emitting part of it as fluorescence. This fluorescence signal, primarily concentrated in the 680–750 nm (red) and 750–800 nm (near-infrared) bands, is considered a direct indicator of plant photosynthetic efficiency (Wu et al., 2024). Although SIF signals are feeble, they can be precisely captured using modern hyperspectral sensors and spectrophotometers (Liu et al., 2020). Hyperspectral resolution typically ranges from 0.3 to 1 nanometer, ensuring the ability to distinguish fluorescence signals from solar scatter interference (Meroni et al., 2009). SIF measurements are not limited to ground observations; they can also be conducted via drones, aircraft, and satellite platforms (Miyauchi et al., 2025). The spatial resolution of satellite observations typically ranges from several hundred meters to several kilometers, while ground observations can achieve resolutions down to a few meters (Joiner et al., 2012). There is a strong correlation between SIF and photosynthetic efficiency, with correlation coefficients typically ranging from 0.6 to 0.9 (Tao et al., 2024). Therefore, SIF is widely used to assess the efficiency of plant photosynthesis, particularly in fields such as ecology, agricultural science, and global environmental monitoring (Li et al., 2018).

In global carbon cycle research, SIF is commonly used to estimate the Gross Primary Productivity (GPP) of plants, which is a crucial indicator of an ecosystem’s carbon absorption capacity (Sun et al., 2017). For instance, Europe’s Fluorescence Explorer (FLEX) satellite project is specifically designed to measure SIF signals globally to enhance understanding of the global carbon cycle (Köhler et al., 2018). By monitoring SIF in real-time, vital information can be obtained about the physiological state of plants under different environmental conditions, providing a scientific basis for crop management and ecosystem protection (Yang, 2019). However, current research on the application of SIF in monitoring soil salinization in the arid regions of Central Asia is still relatively limited. Existing research, such as that by (Du et al., 2024), which utilizes SIF time-series data to build soil salinity models, has achieved some success. However, due to the sparse vegetation in the arid regions of Central Asia, the weak and unstable SIF signals limit the accuracy of monitoring. Therefore, this study aims to fill the research gap in SIF and soil salinization monitoring in Central Asia and Xinjiang. Specific objectives include: (1)Construct spatial zoning and high-precision modeling: The study area is divided using a 1°×1° grid, and typical and atypical regions are identified by analyzing the significance of the relationship between SIF and soil salinity. Based on this, the SIFI modeling method is improved by extending the SIF time series to a full year (January–December), and a high-accuracy soil salinity prediction model is developed for typical regions. The model is then transferred to atypical regions through transfer learning to assess spatial differences across regions.(2)Conduct temporal sensitivity analysis: Long-term (monthly) SIF data are used to evaluate the sensitivity of vegetation fluorescence responses to soil salinity dynamics across different months in the five Central Asian countries and the Xinjiang region of China.(3)Analyze sensitivity variations across land-use types: Land-use classification data are incorporated to assess how SIF responds to soil salinization under different land-use types, thereby enhancing the ecological interpretability of the model.(4)Retrieve regional soil salinization patterns: Based on the SIF–soil salinity models developed for typical and atypical regions, spatial distribution maps of soil salinity are constructed for the entire Central Asia and Xinjiang regions, revealing inter-country and intra-regional differences and temporal trends in salinization.(5)Evaluate observational and modeling limitations: The contribution of monthly SIFI indices to model accuracy is analyzed, along with the limitations imposed by the current spatial resolution of SIF data in monitoring soil salinization, offering insights for future sensor development and monitoring strategies.

## 2 Materials and methods

### 2.1 Study area

**Figure 1:**
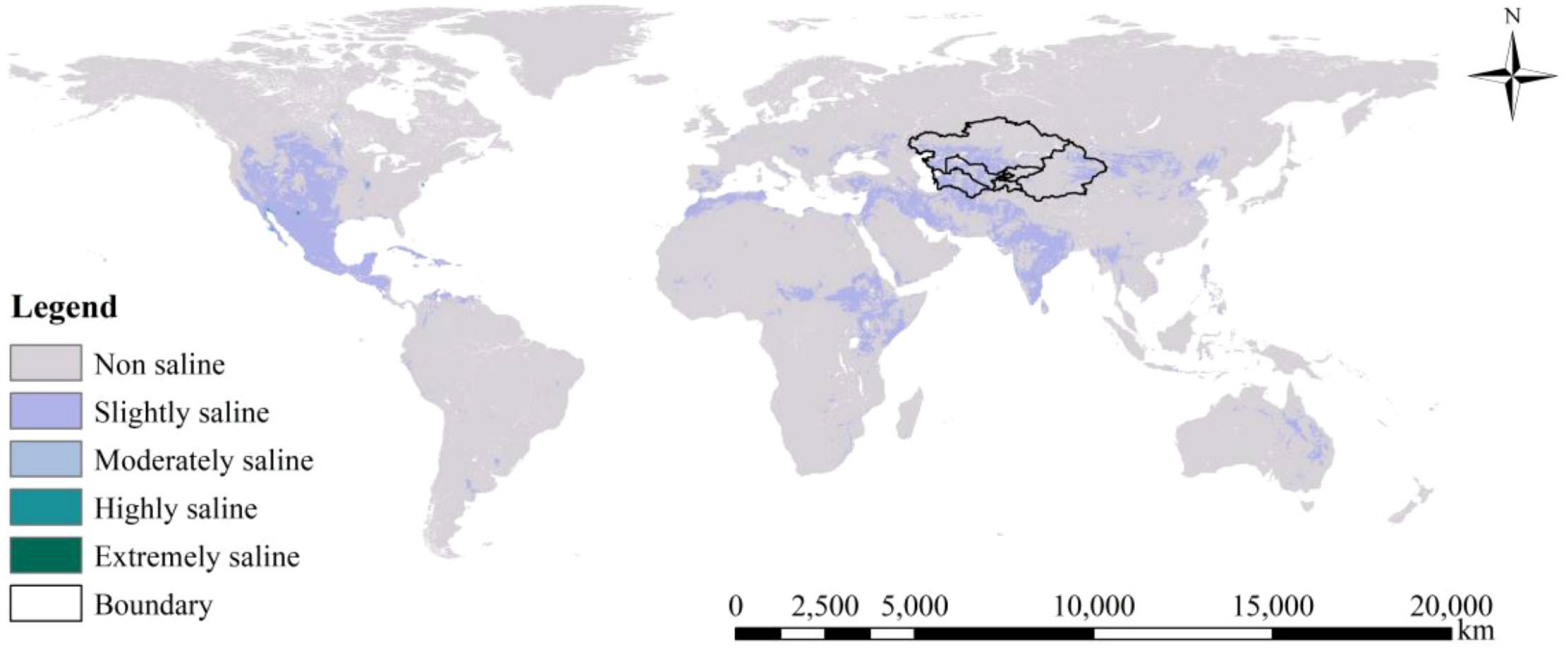
Location of the study area.

Due to their unique geographical and climatic conditions, Central Asia and China’s Xinjiang region face serious soil salinization issues (Jiang et al., 2019). The study area selected for this thesis includes the five Central Asian countries and the Xinjiang Province of China, covering latitudes from 55°27′40″N to 34°20′10″N and longitudes from 46°29′30″E to 96°23′35″E, as shown in **Figure 1**. Due to their unique geographical and climatic conditions, Central Asia and the Xinjiang region face serious soil salinization issues. Central Asia, including Kazakhstan, Uzbekistan, Turkmenistan, Tajikistan, and Kyrgyzstan, features a typical continental climate with vast plains and deserts. This results in higher evaporation than precipitation, high groundwater levels, and significant salt accumulation. The region predominantly comprises extensive plains and deserts, with the plains mainly located in the Syr Darya and Amu Darya river basins, and the deserts spread across the southern and eastern parts of Central Asia (Hamidov et al., 2016). Xinjiang, located in northwestern China, has an arid climate and complex terrain. The natural conditions, including the Tianshan and Kunlun mountain ranges and the vast Taklamakan Desert, further exacerbate soil salinization (Zhuang et al., 2021). Xinjiang is characterized by mountains, basins, and deserts, with the Tianshan and Kunlun Mountain ranges stretching from east to west. The Tarim Basin lies in the central part, which contains the vast Taklamakan Desert (Duan et al., 2022b). Irrational irrigation and drainage systems have exacerbated this issue, leading to reduced crop yields, soil structure degradation, and deterioration of the ecological environment, thereby affecting regional economic and sustainable agricultural development (Wang et al., 2019). Therefore, researching the causes, current status, and prevention measures of soil salinization in these areas is of significant importance for enhancing agricultural productivity and the quality of the ecological environment (Peng et al., 2019).

### 2.2 Data collection

#### 2.2.1 SIF observation dataset

Zhang (2018) utilized OCO-2, the Moderate Resolution Imaging Spectroradiometer (MODIS), and meteorological analysis data (Zhang et al., 2018) to develop a global 0.05°Solar-Induced Chlorophyll Fluorescence (CSIF) dataset based on OCO-2. This dataset was generated using a neural network model built with MODIS channel reflectance and SIF observation data, producing SIF data with high temporal and spatial resolution. The temporal resolution of the data is every four days, and the spatial resolution is 0.05°. Good estimation results were obtained. Therefore, the SIF time series provided by this dataset serves as the data source for solar-induced chlorophyll fluorescence observations in this paper.

#### 2.2.2 Soil salt content

The WoSIS soil profile database contains over 100,000 georeferenced soil profiles, serving as authentic reference data for soil salinity. We selected the upper soil layer (0-10cm) where salinity data was available and classified soil salinity into five levels: non-saline (EC< 2 dSm^−1^), slightly saline (EC = 2 ~ 4 dSm^−1^), moderately saline (EC = 4 ~ 8 dSm^−1^), highly saline (EC = 8 ~ 16 dSm^−1^), and extremely saline (EC > 16 dSm^−1^). We defined soils with EC ≥ 2 ~4 dSm^-1^ as salt-affected soils and those with EC ≥ 4~8 dSm^−1^ as saline soils. Ivushkin et al. (2019) used the SoilGrids global soil category and attribute map dataset and the thermal infrared bands of Landsat remote sensing images as features, with soil salinity samples from the WoSIS soil profile database serving as ground truth. They created global soil salinity classification maps using the random forest classification method on Google Earth Engine (GEE). In this study, the maps produced by Ivushkin et al. (2019) and the WoSIS soil profile database are used as data sources for global soil salinity information. However, WoSIS data is typically sampled annually, whereas CSIF data has a four-day temporal resolution. This inconsistency in time scales may affect data matching and analysis. To address this challenge, we aggregated the original CSIF data annually, calculating yearly CSIF values to ensure consistency between WoSIS sampling times and CSIF acquisition times.

#### 2.2.3 Surface land-use type

When building soil salinity models, it is crucial to consider changes in land use data, as changes in land use patterns directly affect the physical structure and chemical properties of soil. For example, the increase in agricultural activities can lead to higher irrigation water use, which in turn influences the accumulation and distribution of soil salinity. In urbanization, changes in land cover, such as the reduction of vegetation cover and the increase of impervious surfaces, can also alter surface water runoff and evaporation characteristics, thereby affecting the soil’s water and salt balance. We conducted a comparative analysis of the model results based on different land use types to explore the variations in the relationship between Solar-Induced Chlorophyll Fluorescence (SIF) and soil salinity under different land use conditions. The GLC_FCS30D dataset represents a groundbreaking development in global land cover monitoring, offering comprehensive insights into land cover dynamics from 1985 to 2022 with a resolution of 30 meters. The GLC_FCS30D was developed using a continuous change detection method, leveraging the extensive archive of land satellite images available on the Google Earth Engine platform. This dataset achieved high confidence in accuracy, validated by more than 84,000 global samples, with an overall accuracy rate of 80.88% (Zhang et al., 2024b). Twelve major land use types were summarized in **Table 1** to simplify the model-building process.

**Table 1:** The main land use types from the GLC_FCS30D dataset.

Land use	Land classification system	LC id
Rainfed cropland	Rainfed cropland	10
Herbaceous cover	Herbaceous cover	11
Irrigated cropland	Irrigated cropland	20
Forest	Open evergreen broadleaved forest	51
	Closed evergreen broadleaved forest	52
	Open deciduous broadleaved forest	61
	Closed deciduous broadleaved forest	62
	Open evergreen needle-leaved forest	71
	Closed evergreen needle-leaved forest	72
	Open deciduous needle-leaved forest	81
	Closed deciduous needle-leaved forest	82
	Open mixed-leaf forest (broadleaved and needle-leaved)	91
	Closed mixed leaf forest (broadleaved and needle-leaved)	92
Shrubland	Shrubland	120
	Evergreen shrubland	121
	Deciduous shrubland	122
Grassland	Grassland	130
Sparse vegetation	Sparse vegetation	150
	Sparse shrubland	152
	Sparse herbaceous	153
Wetlands	Swamp	181
	Marsh	182
	Flooded flat	183
	Saline	184
	Mangrove	185
	Salt marsh	186
	Tidal flat	187

### 2.3 Sensitivity of solar-induced chlorophyll fluorescence to soil salinity

When vegetation is subjected to soil salinity stress, it is generally believed that the plant’s photosynthetic capacity decreases, and the SIF value correspondingly decreases (Song et al., 2018). However, the corresponding SIF value may not necessarily show significant differences when soil salinity changes significantly over time. Phenological changes, vegetation type, climatic conditions such as precipitation and temperature, and soil characteristics such as moisture and texture are also important drivers of SIF variation (Hamidov et al., 2016). Therefore, it is necessary to filter the samples to eliminate the influence of other factors on SIF and ensure that soil salinity is the dominant factor in SIF observations. Specifically, to avoid the impact of land use changes on SIF, we excluded samples where land use types changed during the study period (2000-2020). Next, we collected samples at 0.05-degree intervals and extracted the corresponding SIF observation values. Finally, we used multiple comparisons to test whether there were significant differences (p< 0.05) in SIF observations under different salinity conditions.

### 2.4 Improved standardized solar induced chlorophyll fluorescence index

Du et al. (2024) developed a method to standardize SIF observations based on time-series data (2000-2020) and probability distribution functions. The standardized Solar-Induced Chlorophyll Fluorescence Index (SIFI) was calculated from the time series of SIFI at different temporal scales. This SIFI was then used to develop a soil salinization model. This standardization method was initially used to normalize precipitation indices to characterize meteorological droughts. In this study, the improved standardized Solar-Induced Chlorophyll Fluorescence Index (SIFI) was calculated from the SIF observation time series at a monthly scale from January to December. The calculation method for the improved SIFI is as follows:

First, the SIF time-series data is fitted to a probability distribution, in this case, a Gamma distribution, to account for its non-negativity and skewness. The probability density function of the Gamma distribution is defined as:


$$f\left(x;\alpha ,\beta \right)=\frac{x^{\alpha -1}e^{-x/\beta }}{\Gamma \left(\alpha \right)\beta^{\alpha }}$$

Here, *x* represents the SIF value, while α and β are the shape and scale parameters, respectively, and Γ is the Gamma function. The parameters α and β are obtained through maximum likelihood estimation (MLE) from the data. After estimating the parameters, the cumulative distribution function (CDF) of the Gamma distribution is calculated, yielding the cumulative probability *G*(*x*) for each SIF observation:


$$G\left(x;\alpha ,\beta \right)=\int_{0}^{x}\frac{t^{\alpha -1}e^{-t/\beta }}{\Gamma \left(\alpha \right)\beta^{\alpha }}dt$$

Next, the cumulative probability *G*(*x*) is transformed into the corresponding quantile of the standard normal distribution, which gives the SIFI value:


$$SIFI_{i}=\Phi^{-1}\left(G\left(x\right)\right)$$

The subscript *i* represents the time scale of SIFI. For example, SIFI1, SIFI2, and SIFI6 correspond to SIFI calculated from SIF observations over time scales of one month, two months, and six months, respectively.

### 2.5 Estimation of soil salinity in arid regions

#### 2.5.1 Regional constraint modeling strategy

**Figure 2:**
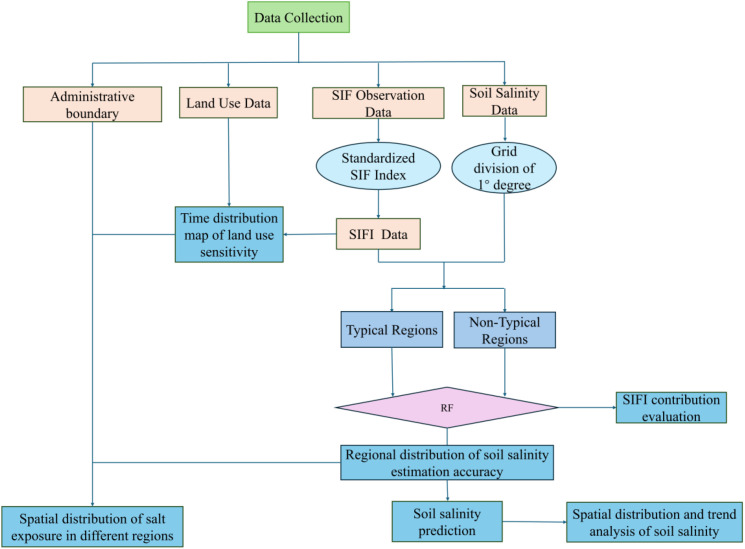
Research workflow diagram.

In this study, the region was divided using a 1°×1° grid, and cross-validation of sample point data within the regions was conducted to determine the regional types. The specific method included dividing the regions based on latitude and longitude information and assigning each number. A two-sample t-test was used to compare the significance of point values within each region, marking them as typical or atypical regions. The results showed that 264 regions were identified as typical (p<0.05), and 464 regions were classified as atypical, containing 83,659 typical sample points and 133,616 atypical sample points. This method ensures a scientific regional division and provides fundamental data support for the development of the soil salinization model, as illustrated in the overall workflow shown in **Figure 2**.

#### 2.5.2 Typical regional models

The model is constructed separately for each specific region, with inputs consisting of combinations of typical sample point SIFI values at different time scales. The number of input variables can vary between 2 and 10. The model output is soil salinity. The highest model accuracy determines the specific number of input variables (i.e., SIFI combinations) to ensure that the selection of inputs is based on performance optimization. We used a random forest classification algorithm to construct the soil salinity model to improve prediction accuracy and model efficiency. During the modeling process, we used GridSearchCV to systematically search for the optimal model parameters, including the number of trees, feature selection range, and maximum tree depth. GridSearchCV evaluates the effectiveness of various parameter combinations through cross-validation, ensuring the identification of the parameter configuration that yields the highest accuracy. Additionally, we introduced regularization techniques to control model complexity and prevent overfitting. By adjusting model parameters, such as maximum tree depth and the maximum number of features, we can limit the model’s learning capacity, enabling it not only to fit the details of the training data but also to generalize to new data.

#### 2.5.3 Atypical regional models

SIF observations under different soil salinity levels did not show significant differences in atypical regions. To address this challenge, we adopted a transfer learning approach. Transfer learning allows us to leverage knowledge from related tasks to solve new problems. Specifically, we selected a pre-trained model with high estimation accuracy in typical regions to estimate soil salinity. The selection was based on the model’s accuracy in estimating soil salinity in the typical areas. The selected optimal typical-region model was adjusted using transfer learning to better adapt its parameters to the environmental characteristics of atypical regions.

## 3 Results

### 3.1 Sensitivity distribution of SIF values to soil salinity in Central Asia and Xinjiang, China

**Figure 3:**
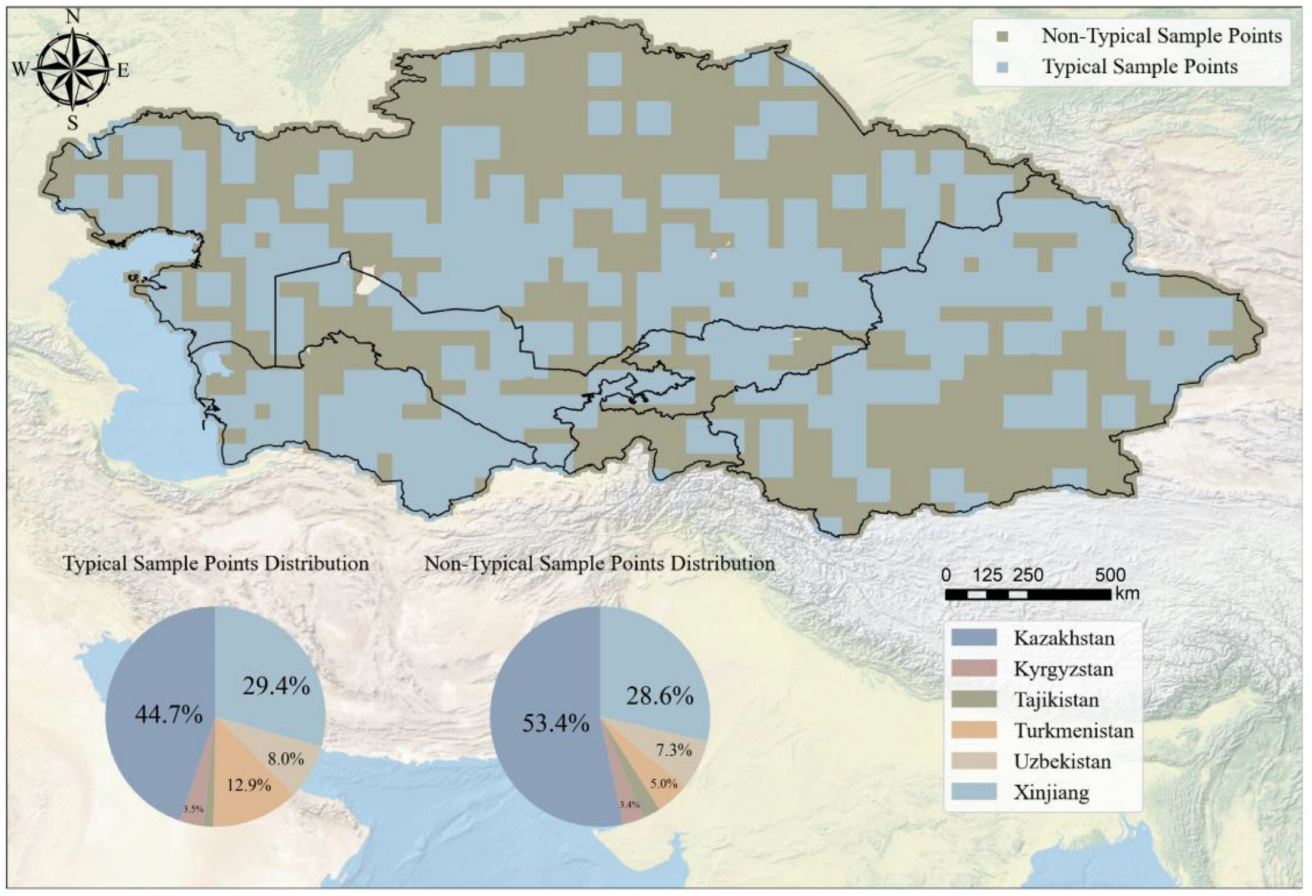
Sensitivity distribution of SIF observation to soil salinity.

We used satellite-based Solar-Induced Chlorophyll Fluorescence (CSIF) observation data to perform sensitivity analysis on all sample points. **Figure 3** shows the sensitivity of SIF values to soil salinity in the five Central Asian countries and the Xinjiang region of China. The results show that 44.7% of the sensitive points were typical sample points in Kazakhstan, indicating many susceptible sample points in this region, followed by Xinjiang, which accounted for 29.4%. In contrast, the proportion of typical sample points in Turkmenistan, Uzbekistan, Kyrgyzstan, and Tajikistan was 12.9%, 8%, 3.5%, and 1.5%, respectively. Non-Typical Sample Points were primarily distributed in Kazakhstan (53.4%) and Xinjiang (28.6%). Spatial distribution analysis indicates that typical sample points in Kazakhstan are predominantly concentrated in its southern region, while those in China’s Xinjiang are primarily located in the northern areas. This distribution pattern may be associated with the spatial extent of core irrigated agricultural zones in both regions: Southern Kazakhstan and Northern Xinjiang represent significant agricultural hubs within their respective territories. Large-scale irrigation under arid/semi-arid climatic conditions may contribute to the development of secondary soil salinization, potentially resulting in heightened sensitivity of vegetation photosynthesis to soil salinity changes in these areas. Turkmenistan exhibits a significantly higher number of typical sample points compared to non-typical points, suggesting a potentially strong association between SIF values and soil salinity in this region, with the model demonstrating relatively favorable performance here. This observation may reflect Turkmenistan’s distinctive environmental context—characterized by extreme aridity intense evapotranspiration, and an irrigated oasis agriculture system. These factors collectively may drive widespread and severe soil salinization. Within relatively sparse vegetation and potentially less complex environmental stress regimes in desert/saline-alkali landscapes, SIF signals may experience reduced interference from confounding biophysical factors. Consequently, the SIF response to soil salinity—as a key stressor—might appear more discernible, potentially enhancing model performance. Conversely, Tajikistan shows a higher proportion of non-typical sample points, and the statistical correlation between SIF values and soil salinity is markedly weaker than in other study regions. This indicates that the SIF response to soil salinity variations may be relatively weak or non-significant in this area. Potential underlying reasons for this discrepancy may lie in Tajikistan’s complex mountainous terrain: Fragmented topography drives substantial heterogeneity in hydrothermal conditions and diverse local microclimates. Vegetation growth and associated SIF signals may be predominantly influenced by, or interact strongly with, topography-controlled water availability, temperature stress, variations in vegetation community types, or other environmental factors not fully quantified. The presence of these complex factors may partially explain the weaker-than-expected association between observed SIF values and soil salinity, posing challenges to model applicability in this region.

### 3.2 Time distribution of SIF sensitivity to soil salinity

**Figure 4:**
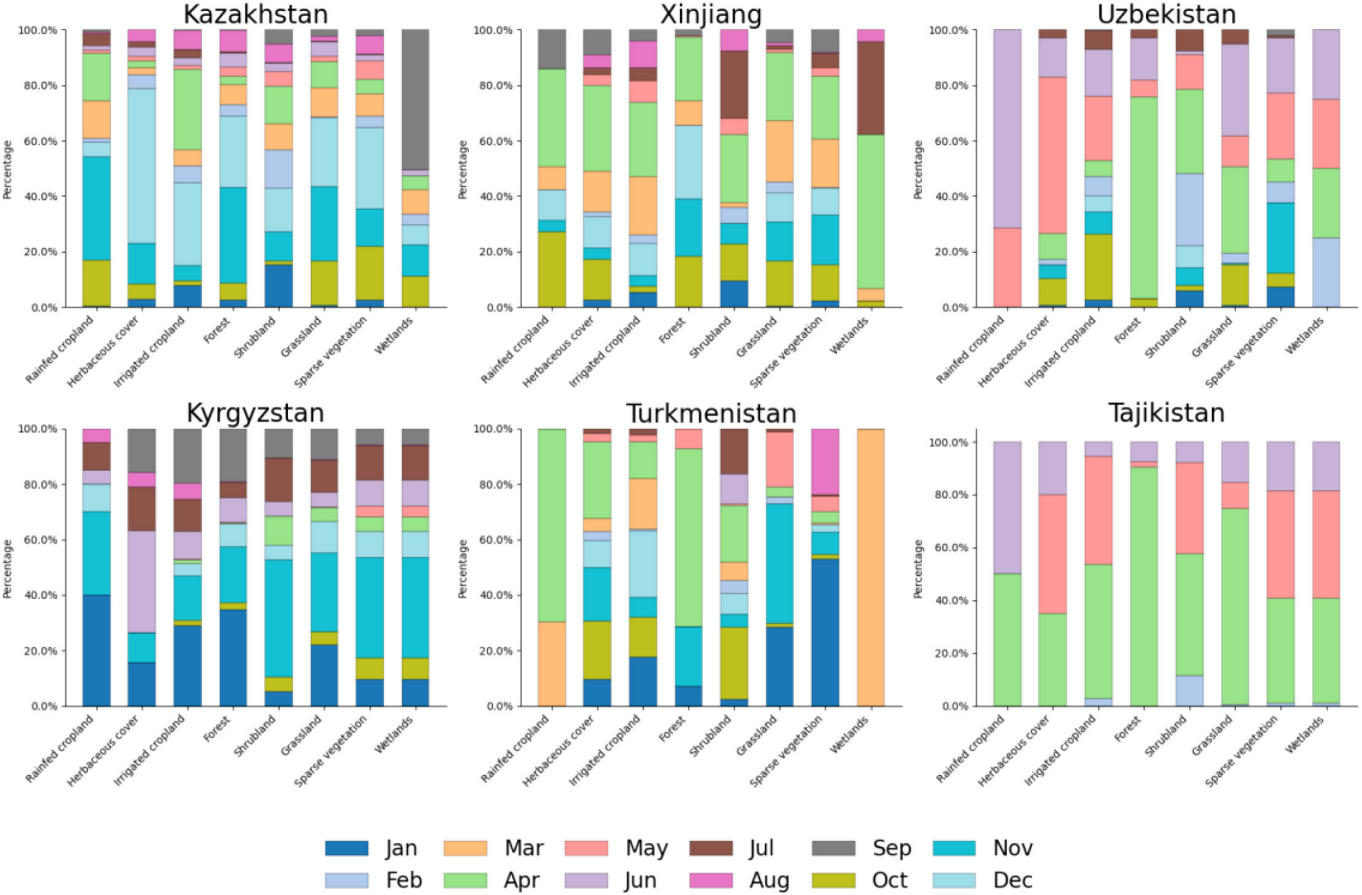
Months sensitive to soil salinity observed by SIF in various regions.

**Figure 4** reveals significant temporal differences in the sensitivity of SIF to soil salinity across the five Central Asian countries and Xinjiang, China, which are closely related to seasonal climate conditions and land-use activities in each region. We found that April is the most sensitive month for all countries. This suggests that in spring, the response of land-use types to changes in soil salinity and model accuracy is most pronounced because increasing temperatures and moisture availability may enhance soil salinity effects, making them easier to detect. Additionally, spring is typically a period of increased agricultural activity, with environmental changes driven by both natural and human-induced factors, making SIF data more sensitive to salinity changes during this month. April was the most sensitive month in Xinjiang, Turkmenistan, and Tajikistan, demonstrating that spring plays a crucial role in land-use types in these regions. In contrast, Kazakhstan was most sensitive in December, Uzbekistan in June, and Kyrgyzstan in November. These differences reflect each country’s specific land-use patterns and climate condition changes during these months. For example, the sensitivity in Uzbekistan in June may be related to higher temperatures and soil moisture variations in early summer, while the high sensitivity in Kazakhstan in December may be linked to cold-season moisture retention and salinity accumulation before deep winter. The distribution of sensitive months also showed significant differences across various terrain types. In Kazakhstan, rainfed cropland, forest, and grassland were most sensitive in November, while herbaceous cover, irrigated cropland, and shrubland showed the highest sensitivity in December, and wetlands peaked in September. This illustrates the different response characteristics of terrain types to seasonal changes, reflecting their varying adaptability to environmental conditions. In Xinjiang, most terrain types, including rainfed cropland, herbaceous cover, irrigated cropland, shrubland, grassland, sparse vegetation, and wetlands, showed peak sensitivity in April, while only forests were most sensitive in December. This consistent sensitivity pattern suggests that April is a critical transition period for soil moisture and salinity effects in Xinjiang. Rainfed cropland in Uzbekistan was most sensitive in June, while herbaceous cover peaked in May, irrigated cropland in October, and forest in April. Shrubland and grassland exhibited peak sensitivity in April and June, respectively, while sparse vegetation and wetlands were most sensitive in November and February. These variations indicate that seasonal changes in soil moisture and vegetation growth cycles significantly impact salinity sensitivity. In Kyrgyzstan, the overall peak sensitivity occurred in November. However, different land cover types exhibited varied sensitivity peaks: rainfed cropland, irrigated cropland, and forest were most sensitive in January, herbaceous cover in June, and shrubland, grassland, sparse vegetation, and wetlands in November. These patterns suggest that the interactions between soil salinity and environmental factors are strongly influenced by winter conditions and early growing season changes. In Turkmenistan, the sensitivity peak in April was particularly notable in rainfed cropland, herbaceous cover, and forest, while irrigated cropland was most sensitive in December, shrubland in October, grassland in November, sparse vegetation in January, and wetlands in March. These results indicate that salinity fluctuations in different land cover types are influenced by distinct seasonal processes, including irrigation patterns, evaporation rates, and precipitation regimes. In Tajikistan, most land cover types, including rainfed cropland, irrigated cropland, forest, shrubland, and grassland, exhibited peak sensitivity in April. In contrast, herbaceous cover, sparse vegetation, and wetlands showed the highest sensitivity in May. These findings suggest that spring is a critical period for soil salinity dynamics in Tajikistan, likely due to increased precipitation, snowmelt, and early agricultural activities.

### 3.3 Regional distribution of soil salinity estimation accuracy

**Figure 5:**
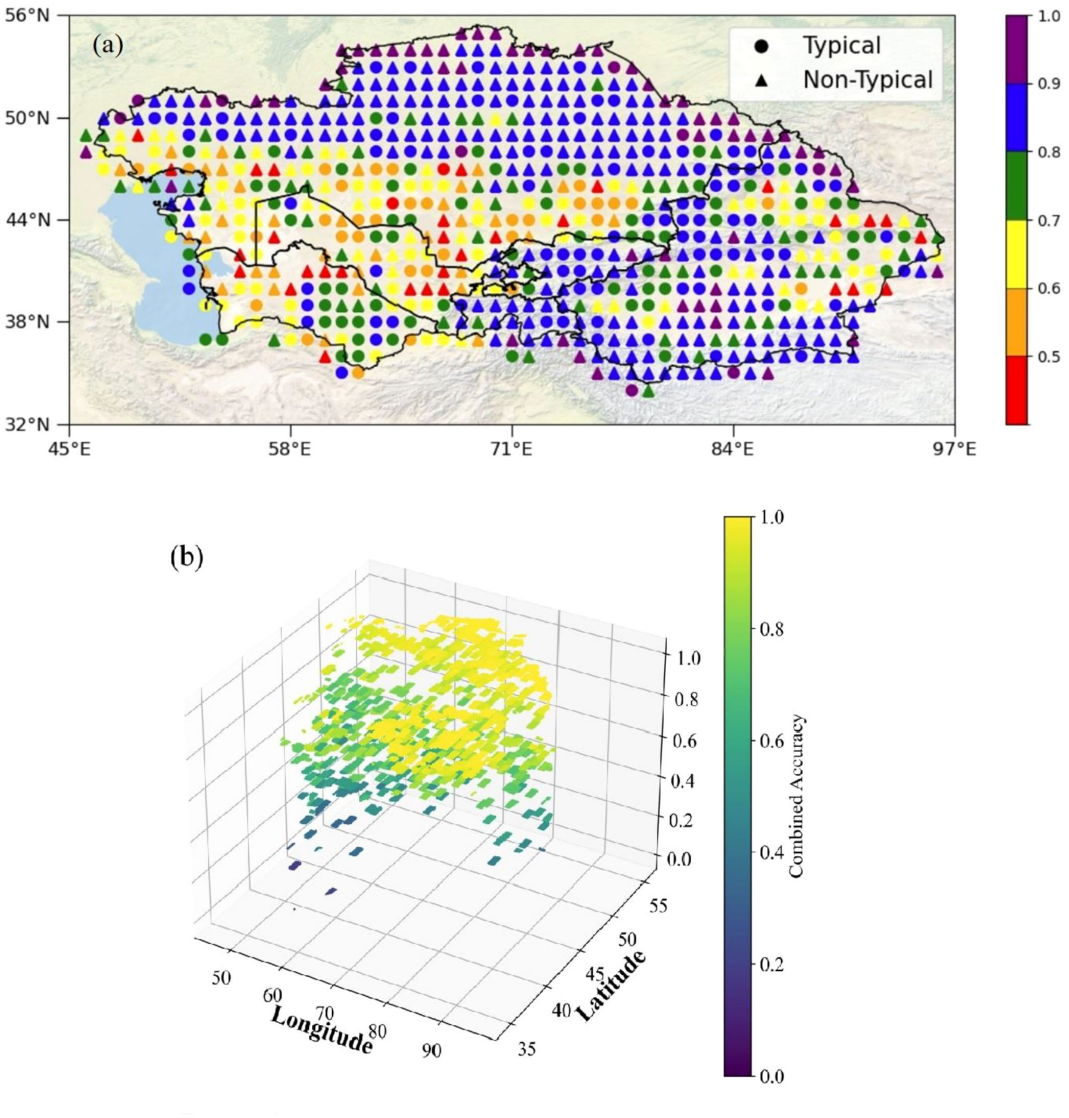
**(a)** Accuracy distribution map of typical and atypical regions **(b)** Statistical scatter plot of accuracy in typical and atypical regions.

(**Figure 5a**) shows the distribution of classification accuracy in different regions for 2016 based on Solar-Induced Chlorophyll Fluorescence (SIF) data. The study found that in most regions, the classification models exhibited high accuracy, further validating the effectiveness and reliability of SIF technology in large-scale soil salinity monitoring. Regarding classification accuracy in typical regions, the model performed well in Tajikistan, which had the highest model accuracy, followed by Kyrgyzstan and Xinjiang, both of which also demonstrated high accuracy. This indicates that SIF data can effectively distinguish the spatial variability of soil salinity in these regions, and the models in these areas have predictive solid capabilities when dealing with typical samples. In contrast, the classification model accuracy in typical regions of Uzbekistan and Turkmenistan was relatively lower. This is related to the diversity of soil types, vegetation cover, and the complexity of land-use patterns in these regions, which resulted in more significant classification errors. In non-typical regions, Kyrgyzstan and Tajikistan again demonstrated high classification accuracy, particularly when handling complex environmental samples, showcasing their models’ strong adaptability and reliability. Xinjiang also performed well, indicating that SIF data in non-typical regions of this area had high monitoring capabilities. However, Turkmenistan’s classification accuracy in non-typical regions was significantly lower, indicating that the model in this region has apparent deficiencies when addressing complex environmental changes, requiring further parameter optimization or the integration of other data sources to improve accuracy. (**Figure 5b**) shows the 3D scatter plot distribution of overall accuracy in various regions. The study found that the spatial distribution of accuracy exhibits some geographical variability. Overall, areas with higher classification accuracy were mainly concentrated in Xinjiang, Tajikistan, and Kyrgyzstan, while regions with lower accuracy were primarily distributed in Turkmenistan and Uzbekistan.

**Figure 6:**
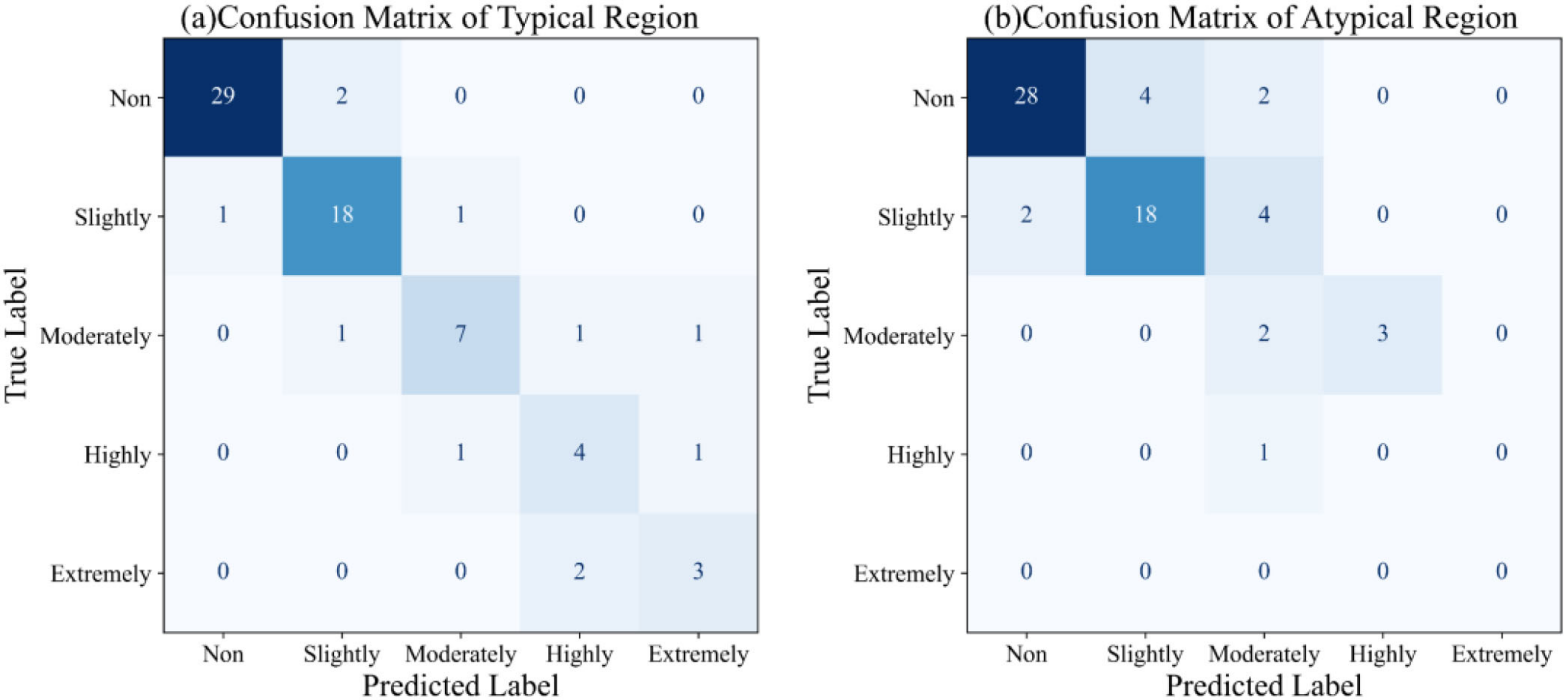
Confusion matrices of the classification results. **(a)** typical Region **(b)** Atypical Region.

This study evaluated the accuracy of classification models for typical and non-typical regions in the five Central Asian countries (Kazakhstan, Kyrgyzstan, Tajikistan, Turkmenistan, and Uzbekistan) and the Xinjiang region of China. By analyzing the 2016 regional divisions and model accuracy data, the median classification accuracy for each region was calculated, allowing for a comparison of model performance across the countries. Due to the imbalance in salinity categories during the modeling process, the estimated models produced more conservative predictions. Most regional models exhibited high classification accuracy, indicating that SIF technology can effectively monitor changes in soil salinity. This result suggests that SIF can be a reliable remote sensing tool for large-scale soil salinity monitoring. The classification accuracy rankings for typical regions were as follows: Tajikistan > Kyrgyzstan > Xinjiang > Kazakhstan > Turkmenistan > Uzbekistan. This indicates that the classification models for Tajikistan, Kyrgyzstan, and Xinjiang performed well in typical regions, exhibiting high accuracy, while the classification performance in Uzbekistan and Turkmenistan needs improvement. The classification accuracy rankings for non-typical regions were Kyrgyzstan > Tajikistan > Xinjiang > Kazakhstan > Uzbekistan > Turkmenistan. Kyrgyzstan and Tajikistan performed exceptionally well in the non-typical areas, showing that their models have high accuracy when dealing with complex environments. In contrast, the classification accuracy in the non-typical Turkmenistan regions was significantly lower, indicating substantial deficiencies in the model’s classification performance in this region. We further analyzed the model’s performance across typical and atypical regions. A comparative analysis of **Tables 2** and **3** reveals that the soil salinity classification model based on solar-induced chlorophyll fluorescence (SIF) demonstrates robust performance in typical sample regions. In particular, the F1-scores for the “Non” and “Slightly” saline categories reached 0.95 and 0.88, respectively, with an overall accuracy of 0.85. These results suggest that the model can effectively capture the nonlinear relationship between SIF signals and soil salinity levels in areas with stable spectral responses and sufficient vegetation cover. In contrast, the classification performance declines notably in atypical sample regions, where the overall accuracy drops to 0.75, and the F1-scores for the “Moderately,” “Highly,” and “Extremely” saline classes are significantly lower. This performance degradation can be attributed to the complex environmental conditions in atypical areas, including sparse vegetation, heterogeneous soil backgrounds, and frequent fluctuations in soil water and salt dynamics. Moreover, the number of training samples for the “Highly” and “Extremely” saline classes is considerably limited in these regions, which constrains the model’s ability to learn their discriminative features effectively and leads to insufficient recognition accuracy for these minority classes. **Figure 6** provides a visual comparison of the classification results between typical and atypical regions through confusion matrices. As shown in (**Figure 6a**), the majority of samples in the typical region are correctly classified, especially in the “Non” and “Slightly” categories, which exhibit minimal misclassification. In contrast, (**Figure 6b**) illustrates increased confusion among the moderate to extreme salinity classes in the atypical region, confirming the model’s limited ability to generalize under less stable and underrepresented conditions. Nevertheless, the performance gap between typical and atypical regions provides meaningful insights into the model’s reliability and stability. The high accuracy achieved in typical samples indicates that the proposed SIF-based feature framework is capable of delivering accurate predictions in well-structured and representative regions. Meanwhile, the intentional construction of atypical sample sets and the observed performance drop therein serve as a valuable approach to assess the model’s generalization boundaries and application scope. This validation strategy, which incorporates sample representativeness as a key factor, enhances the interpretability of model performance and offers a practical framework for evaluating classification models under complex ecological conditions.

**Table 2:** Classification metrics for typical sample region.

	Non	Slightly	Moderately	Highly	Extremely	Accuracy
F1-score	0.95	0.88	0.74	0.62	0.60	0.85
Precision	0.97	0.86	0.78	0.57	0.60	—
Recall	0.94	0.90	0.70	0.67	0.60	—

**Table 3:** Classification metrics for atypical sample region.

	Non	Slightly	Moderately	Highly	Extremely	Accuracy
F1-score	0.87	0.78	0.29	0.00	0.00	0.75
Precision	0.93	0.82	0.22	0.00	0.00	—
Recall	0.82	0.75	0.40	0.00	0.00	—

### 3.4 Regional distribution of soil salinity in Central Asia and Xinjiang, China

**Figure 7:**
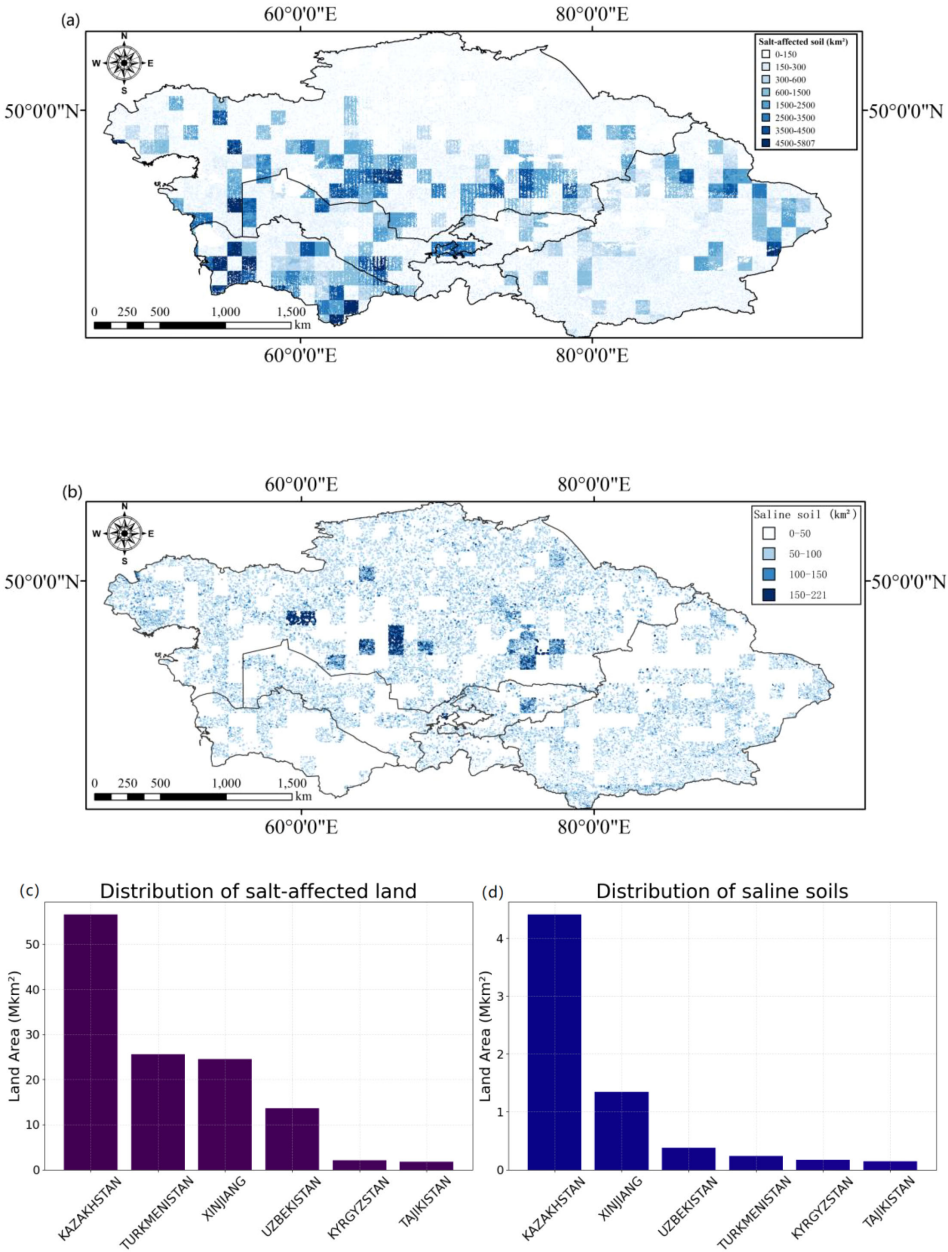
**(a, b)** Distribution of Salt-Affected and Saline soil Land Area **(c, d)** Statistical Chart of Salt Affected Land Area and Salt Affected Land Area.

(**Figures 7a, b**) are generated based on the SIFI index and regional models by analyzing the relationship between SIF data and soil salinity, creating soil salinity distribution maps for salt-affected and saline lands. The results show that Kazakhstan has the largest area of salt-affected and saline lands, particularly in the southern part of the country where irrigation-induced secondary salinization dominates, significantly more than other nations. Additionally, northern Xinjiang characterized by endorheic basins amplifying salt accumulation and southern Turkmenistan exhibiting capillary-driven salt efflorescence also exhibit relatively high areas of salt-affected land. Northern Xinjiang and southern Turkmenistan also have relatively large areas of salt-affected land. SIF observation results can identify the distribution of salt-affected lands (EC ≥ 2~4 dS m^-1^) in Central Asia and Xinjiang demonstrating moderate-salinity detection capability. According to (**Figure 7c**), Kazakhstan has the largest total area of affected saline land, reaching 56.54 Mkm² reflecting widespread irrigation legacy impacts, indicating that salinization is the most severe in this country. Next are Turkmenistan and Xinjiang, with 25.60 Mkm² tied to intensive cotton monoculture and 24.50 Mkm² concentrated in northern piedmont oases respectively. Uzbekistan has 13.66 Mkm² of saline land primarily in the Fergana Valley, while Kyrgyzstan and Tajikistan have relatively more minor areas, with 2.12 Mkm² limited by mountainous drainage and 1.79 Mkm² restricted by steep topography respectively. This distribution difference indicates that Kazakhstan faces the most severe soil salinization problem, affecting a significant portion of its land, whereas Kyrgyzstan and Tajikistan are relatively less affected. (**Figure 7d**) shows the data for saline land (EC ≥ 4~8 dS m^-1^), with Kazakhstan having 4.41 Mkm² of saline land notably near Aral Sea disaster zones, followed by Xinjiang with 1.34 Mkm² associated with paleo-salt deposits and Uzbekistan with 0.38 Mkm². Turkmenistan has 0.23 Mkm² of saline land focused along canal networks, while Kyrgyzstan and Tajikistan have 0.17 Mkm² confined to valley bottoms and 0.15 Mkm² in localized depressions respectively.

### 3.5 Distribution of SIFI contributions to soil models at different time scales

**Figure 8:**
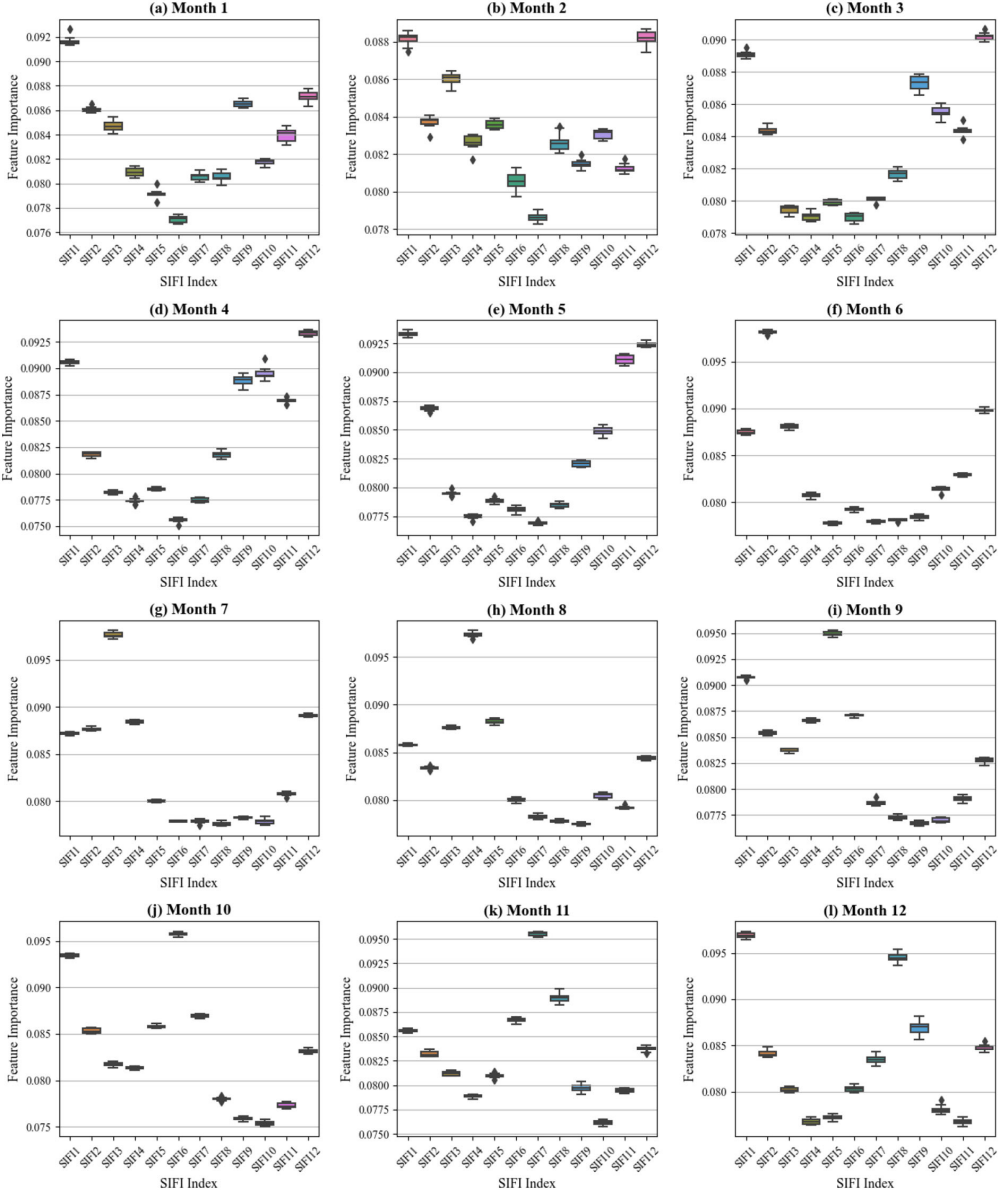
SIFI index’s contribution to the model. **(a-i)** Analysis of Model Contribution from January to December.

The contributions of different SIFI indices to the model vary; therefore, we analyzed the feature importance of each SIFI index across different months to explore its influence over time. This study employed a random forest model to analyze the feature importance of SIFI1-SIFI12 variables across different months, revealing significant differences in predictive capability across temporal scales. As shown in the **Figure 8**, some variables, such as SIFI1 and SIFI12, consistently exhibited high importance throughout the year, suggesting that they may carry stable and critical environmental information and contribute significantly to soil salinity prediction. In contrast, other variables, such as SIFI6 and SIFI8, had relatively low importance in certain months, indicating that their influence might be restricted to specific time windows. Additionally, in months like February, June, and September, some variables exhibited considerable fluctuations in importance, implying that the predictive ability of SIFI indices during these periods could be affected by external environmental factors. Overall, the temporal evolution trend indicates substantial variations in the importance of specific variables across different months. In long-term prediction tasks, it is advisable to prioritize stable key variables that maintain high importance throughout the year while employing time-series-based dynamic modeling strategies for variables whose importance fluctuates over time to optimize predictive performance. Furthermore, the temporal variability in feature importance suggests that SIFI indices may be influenced by seasonal climate conditions or other environmental factors. Future studies could integrate external environmental variables, such as precipitation and temperature, to further investigate their impact on SIFI indices.

## 4 Discussion

### 4.1 Trend distribution/regional differences of SIF values in Central Asia and Xinjiang, China, on soil salinity and soil salinity distribution

**Figure 9:**
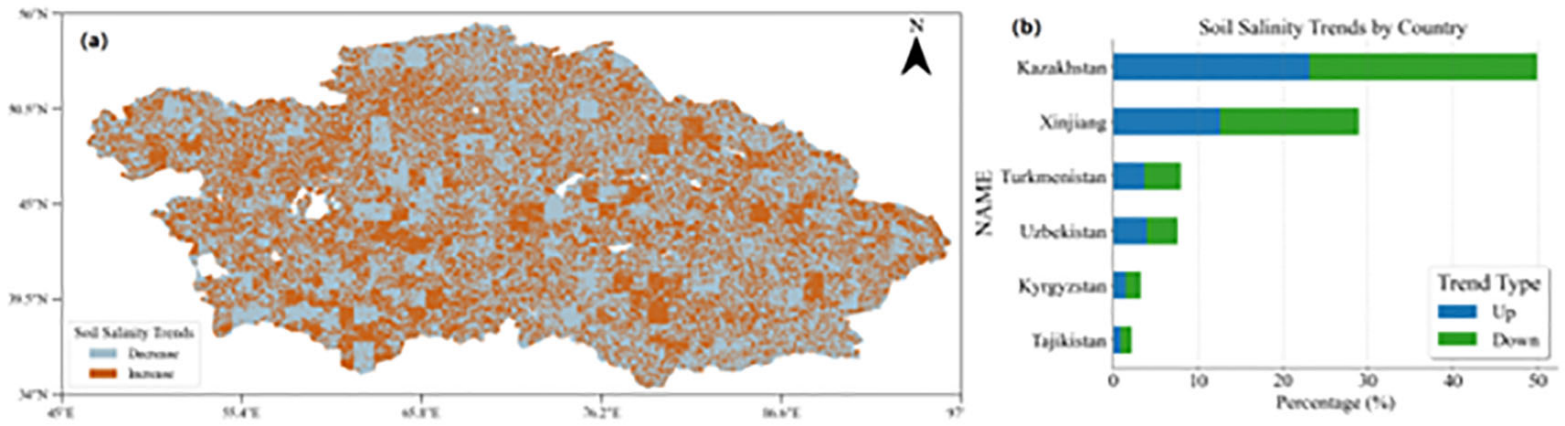
Statistical distribution of soil salinity trend in the study area **(a)** Salinity Trend Distribution Map **(b)** Salinity trend bar chart.

We used Solar-Induced Chlorophyll Fluorescence (SIF) observation data from all sample points in Central Asia and Xinjiang between 2000 and 2020 to create a soil salinity change map for 2000-2020 (**Figure 9**). The results show a significant increase in soil salinity in southern Kazakhstan and around Lake Balkhash, while the salinity in northern Kazakhstan has decreased. The soil salinity in western Xinjiang also showed an increasing trend. This is consistent with the findings of Issanova et al. (Issanova et al., 2017). The increase in soil salinity in southern Kazakhstan and southern Central Asia primarily occurs in bare land, shrubland, and irrigated cropland. These land types are sensitive to environmental changes and prone to salinization. This may be related to reduced local precipitation, increased evaporation, and improper irrigation practices (Lobell et al., 2011). In contrast, the decrease in soil salinity in northern Kazakhstan may reflect improvements in agricultural management practices, such as adopting more efficient irrigation technologies, reducing over-cultivation, and promoting natural vegetation restoration (Zhang et al., 2010). The increasing trend in soil salinity in western Xinjiang may be closely related to agricultural expansion, improper water resource management, and secondary salinization caused by irrigation in the region (Zaitunah et al., 2018). More than 60% of the sample points in Central Asia and Xinjiang show an increasing trend in soil salinity, further confirming that the expansion of saline land has become a widespread phenomenon in these regions. Soil salinization poses a serious threat to agricultural production and may trigger a range of ecological and environmental issues, such as vegetation degradation, soil erosion, and the deterioration of soil structure (Tarolli et al., 2024). These issues are particularly pronounced in arid and semi-arid regions, as soil salinization often exacerbates water scarcity, further impacting regional ecological balance and sustainable development (Doan et al., 2023). The spatiotemporal variation of soil salinity is influenced by multiple factors, including climate change, human activities, and natural geographic features (Haj-Amor et al., 2023). The increase in salinity in southern Kazakhstan and around Lake Balkhash may be related to reduced precipitation and increased evaporation caused by global warming, and it could also be the result of agricultural expansion and improper irrigation (Tarolli et al., 2024). On the other hand, the decline in salinity in northern Kazakhstan may be related to improvements in agricultural management strategies in recent years, such as introducing more scientific irrigation techniques and natural vegetation restoration measures (Rakhmanov et al., 2024). The increase in salinity in western Xinjiang also warrants attention, as this phenomenon may be due to improper water resource management and secondary soil salinization caused by irrigation during the region’s agricultural development (Bai et al., 2023). Therefore, improving irrigation techniques, increasing soil organic matter, promoting salt-tolerant crops, and encouraging vegetation restoration are effective methods to slow the process of salinization (Liu et al., 2022). At the same time, using advanced remote sensing technologies like SIF for long-term monitoring allows for precise tracking of the dynamic changes in soil salinity and provides vital reference data for formulating more scientific and practical soil management policies. This will help ensure the sustainability of regional ecosystems and support the long-term stable development of agricultural production (Aburas et al., 2015).

### 4.2 Comparison of SIF global validation results in Central Asia and Xinjiang, China

**Figure 10:**
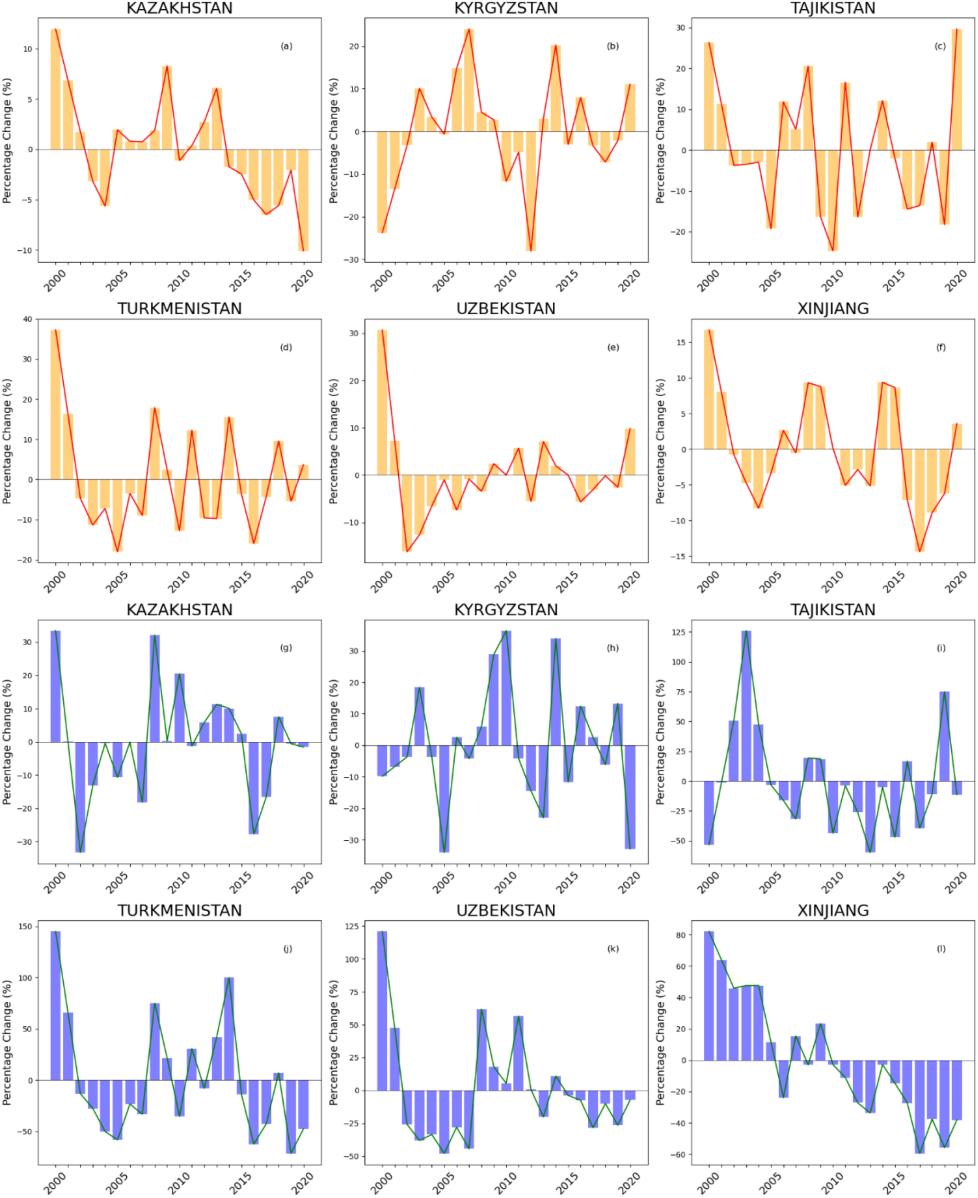
**(a–f)** Spatiotemporal changes in land area affected by salt in various countries, **(e–l)** Spatiotemporal changes in land area affected by salt in various countries.

Using Solar-Induced Chlorophyll Fluorescence (SIF) data from 2000 to 2020, combined with machine learning methods and the Standardized Solar-Induced Chlorophyll Fluorescence Index (SIFI), a systematic analysis of the spatiotemporal changes in soil salinity across the five Central Asian countries and the Xinjiang region of China was conducted. It was found that the increase in soil salinity poses a severe threat to agricultural production, with significant differences in the degree and trends of soil salinization across the countries. The increase in soil salinity is typically negatively correlated with crop growth inhibition (Shi et al., 2023). Optical and thermal remote sensing technologies effectively monitor this process, especially by using data that reflect vegetation biochemical and physical characteristics, such as leaf water content, leaf area, and chlorophyll content, which can indirectly infer soil salinity levels (Jiang and Shu, 2019). Chlorophyll fluorescence technology is particularly effective, as salinity stress directly inhibits plant photosynthesis, affecting the chlorophyll fluorescence signal (Zaghdoudi et al., 2011). Additionally, by measuring the content of ions such as sodium, potassium, and calcium in the leaves, as well as leaf conductivity, the effects of soil salinity on plants can be further reflected and assessed (Munns and Tester, 2008). These physiological response parameters provide direct evidence of plant health and serve as effective indicators for monitoring soil salinization. Between 2000 and 2020, the area of salt-affected land (soil electrical conductivity, EC ≥ 2–4 dS·m⁻¹) in Tajikistan fluctuated significantly, ranging from 20% to 60%, as shown in **Figure 10**. This fluctuation may be closely related to Tajikistan’s climate conditions, land-use patterns, and changes in agricultural management practices (Orlovsky and Orlovsky, 2002). In contrast, the area of salt-affected soil in Xinjiang, China, has remained relatively stable, with fluctuations around 15%. This relative stability in Xinjiang may be attributed to the region’s stricter water resource management and ongoing land improvement measures (Yang et al., 2019). However, the area changes of saline land (EC ≥ 4~8 dSm^−1^) in various countries have been more drastic than in salt-affected land, highlighting the severity of soil salinization in Central Asia and Xinjiang. For example, the fluctuation range of saline land in Tajikistan is between 50% and 150%, far exceeding that of other countries. This may be related to the country’s extreme climatic conditions, limited water resources, and improper irrigation practices (Turdaliev et al., 2023). In contrast, the relatively stable changes in saline land in Kazakhstan may reflect the progress the country has made in recent years in agricultural management and improvements in irrigation techniques (Duan et al., 2022a). Kyrgyzstan and Tajikistan exhibited significant interannual fluctuations, with sharp alternations in the extent of both salt-affected and saline lands, reflecting high sensitivity to climate variability and instability in land use and irrigation management. In particular, Tajikistan showed annual variation rates in saline land exceeding 100% in multiple years, highlighting its high risk under extreme climatic conditions and improper irrigation practices. Uzbekistan experienced considerable fluctuations in the early years of the study period, but gradually showed a trend of stabilization after 2010. In contrast, Turkmenistan continued to exhibit intense year-to-year variability, with changes in saline land reaching up to 150%, likely associated with periodic accumulation and leaching cycles of salt. The Xinjiang region displayed clear cyclical patterns, and since 2010, saline land has steadily decreased, indicating the positive effects of long-term governance and mitigation efforts. These interannual variation patterns are valuable for distinguishing between short-term, climate-driven transient salinization and structural salinization caused by poor water resource management, thus providing a basis for region-specific differentiated control strategies. Overall, from 2000 to 2020, the dynamics of soil salinization in the five Central Asian countries and the Xinjiang region exhibited significant spatial heterogeneity and temporal variability. Countries such as Kazakhstan and Xinjiang have shown a certain degree of effectiveness in governance and trend stability, whereas Tajikistan and Turkmenistan still face frequent fluctuations and high-intensity salinization pressure. Such disparities are closely related not only to differences in water resource regulation, agricultural management, and irrigation efficiency, but also to the long-term impacts of climatic conditions and land use structures. Therefore, when formulating regional soil salinization control strategies, it is essential to fully consider each area’s climatic vulnerability, governance capacity, and the underlying mechanisms of salt accumulation. Tailored, locally adapted management approaches should be adopted to ensure the sustainable use of land resources and the resilient development of agricultural ecosystems.

### 4.3 Land-type-specific contributions of SIFI indicators

**Figure 11:**
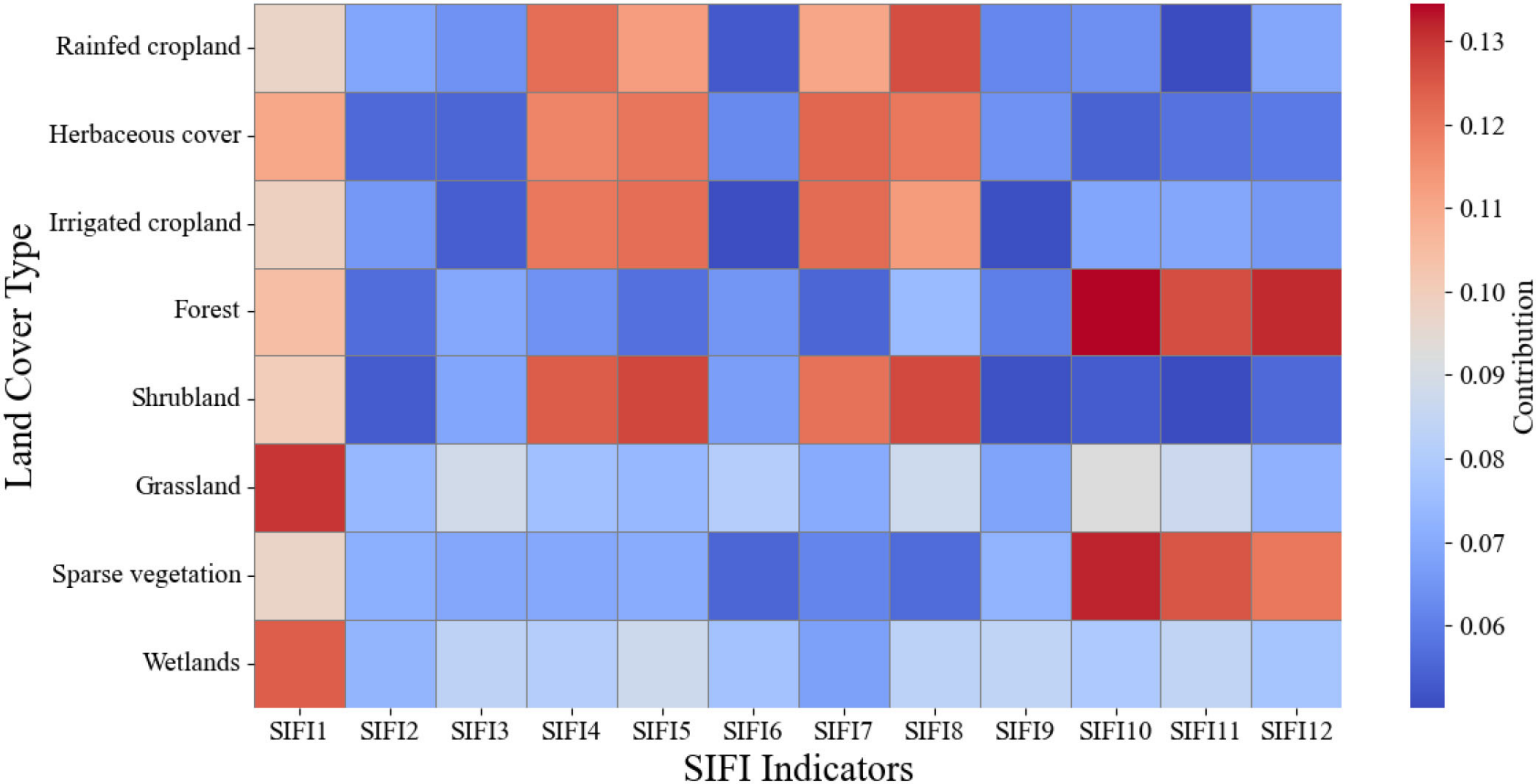
Relative modeling contributions of standardized SIFI indicators across eight representative land cover types.

The modeling contribution of standardized solar-induced chlorophyll fluorescence indices (SIFI1–SIFI12), derived from annual SIF time series, was evaluated across eight representative land cover types. This approach produces phenologically interpretable indicators that characterize the statistical structure of SIF variations and emphasize their differential responsiveness across diverse land cover types, enabling robust comparisons among ecosystems with distinct vegetation characteristics. **Figure 11** reveals that SIFI1 consistently exhibits high contribution across all land cover types, underscoring its robustness as a general sensitivity indicator. Its wide-ranging applicability may stem from its ability to capture early-season physiological activity or reflect persistent structural traits of vegetation that influence SIF dynamics throughout the year. In addition, SIFI4, SIFI5, SIFI7, and SIFI8 show particularly strong contributions in rainfed cropland, herbaceous cover, irrigated cropland, and shrubland. These indices correspond to the mid-growing season, typically associated with peak photosynthetic activity from April to August. During this period, vegetation in these land types is especially responsive to environmental stressors such as salinity, drought, or nutrient limitations, which enhances the explanatory power of SIF-based indicators. In contrast, SIFI10, SIFI11, and SIFI12 exhibit higher contributions in forest and sparse vegetation, indicating their sensitivity to ecological dynamics in late autumn and early winter. This seasonal window is critical for capturing declines in photosynthetic activity, changes in carbon assimilation efficiency, and other long-term traits of perennial or woody vegetation systems.

### 4.4 Study limitations and future work

#### 4.4.1 limitations of spatial resolution

Although this study demonstrates the great potential of SIF technology in soil salinity monitoring, there are still some limitations. For example, current satellite observations of Solar-Induced Chlorophyll Fluorescence (SIF) have certain restrictions in terms of spatial resolution. The resolution of SIF observations typically only reaches the kilometer level, which is insufficient for regions requiring high-precision monitoring, making it challenging to meet the demands for detailed observations. This issue significantly affects our ability to detect and analyze small-scale soil salinity changes accurately. To address this, future research should focus on using new fluorescence satellites with higher resolution, such as those with 300-meter resolution, to conduct experimental studies. This is expected to significantly improve the spatial resolution of soil salinity monitoring and enhance early warning and management of saline land. Additionally, although the random forest model has shown good robustness and accuracy in handling soil salinity monitoring data, it still has limitations when dealing with data from complex terrain or variable climate conditions, which may lead to increased prediction errors (Suleymanov et al., 2023). Therefore, future research should explore more advanced machine learning methods, such as Deep Learning and Support Vector Machines (SVM), to further improve the models’ prediction accuracy and generalization capabilities (Cui et al., 2023). Through their ability to perform automatic feature extraction and nonlinear mapping, these methods can better capture the complex relationships between soil salinity and environmental variables. Additionally, the fusion of multi-source remote sensing data (such as spectral, radar, and thermal infrared data) can provide more comprehensive soil and vegetation information, significantly enhancing the accuracy and reliability of soil salinity monitoring (He et al., 2023). With these technological improvements, future soil salinity monitoring will achieve higher spatiotemporal resolution, more accurate salinity change predictions, and provide a more scientific basis for land management and agricultural decision-making.

#### 4.4.2 Temporal resolution and ground-truthing limitations

Although CSIF, as a high-temporal-resolution solar-induced chlorophyll fluorescence index, can effectively characterize plant photosynthetic activity and short-term stress responses, there exists a significant temporal scale mismatch between CSIF and the WoSIS soil profile data. This inconsistency may introduce systematic bias into model development and interpretation. Specifically, CSIF is acquired at a 4-day interval, reflecting the dynamic and continuous physiological status of vegetation, whereas WoSIS provides soil salinity observations that are mostly based on single, annual-scale sampling events. The temporal information in WoSIS is often coarse, typically limited to the sampling year, which lacks the ability to capture intra-annual variations in soil conditions. This temporal mismatch may introduce uncertainty on several levels. In agricultural systems, especially those with drip irrigation, crop rotation, or multiple cropping practices, the response relationship between soil salinity and SIF signals is often asynchronous. On the one hand, CSIF may increase rapidly following irrigation, rainfall, or short-term climatic fluctuations, capturing the short-term recovery of photosynthetic activity. On the other hand, soil salinity, as a cumulative variable, usually exhibits a delayed and gradual intra-annual response. For example, in typical cotton-growing regions of Xinjiang, CSIF values peak between mid-April and mid-August due to active crop growth and irrigation. During this period, surface SIF may remain high, while the actual soil electrical conductivity (EC) in the upper profile may still indicate moderate or high salinity levels, leading to inconsistencies between remotely sensed indices and measured soil salinity. In natural ecosystems—such as sparse vegetation zones, shrublands, or forest edges—annual soil salinity variation is mainly governed by soil water redistribution, evapotranspiration balance, and root-level ion uptake. The magnitude of such changes is generally small. However, SIF is highly sensitive to plant physiological responses, and even minor water-salt stress may induce noticeable changes in fluorescence intensity. As a result, CSIF in these regions often shows faster and more pronounced variation than the actual soil salinity, further exacerbating the temporal mismatch and potential bias.

## 5 Conclusion

We combined Solar-Induced Chlorophyll Fluorescence (SIF) data, soil salinity observation data, and land-use information to train standardized SIF indices (SIFI) across different time scales using machine learning methods to estimate soil salinity in Central Asia and the Xinjiang region. The main advantage of SIF technology in this experiment is that it can directly reflect the photosynthetic state of plants, which is significantly affected by soil salinity. Therefore, SIF, as an indirect tool for soil salinity detection, demonstrates high sensitivity. In addition, SIF is less affected by atmospheric and soil background interference in soil modeling, making it more suitable for arid regions with sparse vegetation, such as Central Asia and Xinjiang, than traditional remote sensing techniques. By conducting a multi-period analysis of SIF data, we can capture dynamic changes in soil salinity over different time scales, thus constructing a more accurate soil salinity model. Regarding data processing, we used a random forest classification algorithm to analyze SIF data. We determined the optimal response period of SIF to soil salinity changes through sensitivity analysis. The study found that: (1) SIF observations in April showed the highest sensitivity to soil salinity, especially in Kazakhstan and Xinjiang, where the response of SIF data to soil salinity was particularly significant. The model’s classification accuracy in typical regions exceeded 80%, while in non-typical areas, it reached over 70%. (2) The model also revealed the spatiotemporal distribution of soil salinity in the five Central Asian countries and Xinjiang, showing that between 2000 and 2020, salinization was most severe in Kazakhstan and Xinjiang, while soil salinity in Tajikistan fluctuated significantly. (3) The advantages of SIF observations make it more effective than other remote sensing technologies for large-scale soil salinity monitoring, allowing for a better reflection of soil salinity trends in complex environments. In summary, this study validated the effectiveness of SIF technology in large-scale soil salinity monitoring. It demonstrated its scientific application value in addressing salinization issues in Central Asia and Xinjiang. The results provided precise tools for monitoring soil salinity dynamics in regional agricultural production and offered essential references for ecological management and optimal land resource utilization. Future research can further combine higher-resolution SIF data with multi-source remote sensing data to improve the accuracy and applicability of the model.

## Data Availability

The original contributions presented in the study are included in the article/supplementary material. Further inquiries can be directed to the corresponding author.
